# Within-Host Diversity of Coagulase-Negative Staphylococci Resistome from Healthy Pigs and Pig Farmers, with the Detection of *cfr-*Carrying Strains and MDR-*S. borealis*

**DOI:** 10.3390/antibiotics12101505

**Published:** 2023-10-02

**Authors:** Idris Nasir Abdullahi, Carmen Lozano, Carmen Simón, Myriam Zarazaga, Carmen Torres

**Affiliations:** 1 Area of Biochemistry and Molecular Biology, OneHealth-UR Research Group, University of La Rioja, 26006 Logroño, Spain; idris-nasir.abdullahi@unirioja.es (I.N.A.); carmen.lozano@unirioja.es (C.L.); myriam.zarazaga@unirioja.es (M.Z.); 2 Faculty of Veterinary Medicine, University of Zaragoza, 50001 Zaragoza, Spain; mcsimon@unizar.es

**Keywords:** coagulase-negative staphylococci, *Staphylococcus borealis*, multidrug resistance, pig farms, linezolid resistance, *cfr*

## Abstract

The ecology and diversity of resistome in coagulase-negative staphylococci (CoNS) from healthy pigs and pig farmers are rarely available as most studies focused on the livestock-associated methicillin-resistant *S. aureus*. This study aims to characterize the antimicrobial resistance (AMR) mechanisms, intra-host species diversity (more than one species in a host), and intra-species AMR diversity (same species with more than one AMR profile) in CoNS recovered from the nasal cavities of healthy pigs and pig farmers. One-hundred-and-one CoNS strains previously recovered from 40 pigs and 10 pig farmers from four Spanish pig farms were tested to determine their AMR profiles. Non-repetitive strains were selected (*n* = 75) and their AMR genes, SCC*mec* types, and genetic lineages were analyzed by PCR/sequencing. Of the non-repetitive strains, 92% showed a multidrug resistance (MDR) phenotype, and 52% were *mecA*-positive, which were associated with SCC*mec* types V (46.2%), IVb (20.5%), and IVc (5.1%). A total of 28% of the pigs and pig farmers had intra-host species diversity, while 26% had intra-species AMR diversity. High repertoires of AMR genes were detected, including unusual ones such as *tetO*, *ermT*, *erm43*, and *cfr*. Most important was the detection of *cfr* (in *S. saprophyticus* and *S. epidermidis-*ST16) in pigs and pig farmers; whereas MDR*-S. borealis* strains were identified in pig farmers. Pig-to-pig transmission of CoNS with similar AMR genes and SCC*mec* types was detected in 42.5% of pigs. The high level of multidrug, within-host, and intra-species resistome diversity in the nasal CoNS highlights their ability to be AMR gene reservoirs in healthy pigs and pig farmers. The detection of MDR-*S. borealis* and linezolid-resistant strains underscore the need for comprehensive and continuous surveillance of MDR-CoNS at the pig farm level.

## 1. Introduction

Antimicrobial resistance (AMR) is one of the greatest global health threats of the late 21st century [1,2]. The global AMR crisis has persisted mainly due to the transfer of antibiotic-resistant bacteria between animals, humans, and the environment through their shared habitats [3]. The emergence and spread of antibiotic-resistant staphylococci are often blamed on the over-prescription of antibiotics for treatment in humans and animals and as growth enhancers in livestock production [4]. The use of antibiotics as growth enhancers is now banned in many countries, but this is still allowed in others.

Coagulase-negative staphylococci (CoNS) are primarily nasal commensals, although some strains can be opportunistic pathogens; they have been implicated in many infections in humans and animals such as catheter-associated, prosthetic joint, and laryngeal infections or sepsis, among others [5,6]. Recently, new CoNS species have been re-classified. In this regard, it is important to mention the reclassification of *S. borealis* nov. sp., which was previously considered as *S. haemolyicus* [7]. So far, *S. borealis* has been detected in human skin and blood samples [7]. Being a new species, there are no available data on its virulence potential and antimicrobial resistome. The methicillin-resistant trait of CoNS (MRCoNS) has mainly been represented by the emergence and spread of certain multidrug-resistant (MDR) strains with the potential to transfer AMR genes to “more perceived” pathogenic *S. aureus* strains through mobile genetic elements [8]. Some CoNS have been shown to carry the SCC*mec* genetic elements (for *mecA* and *mecC* genes) and plasmids (e.g., *ermT* gene) [8,9]. These mobilome-bound AMR genes could be acquired by certain other *Staphylococcus* species via horizontal transfer [8,9]. Moreover, some CoNS strains can contain critical and transferable linezolid resistance genes [10].

The identification of the source, reservoir hosts, and vectors of transmission of antibiotic-resistant staphylococci can be an arduous task. Food-producing animals may be one of the reservoirs of MRCoNS [11,12]. Although a study had previously revealed the emergence of multiresistance or linezolid resistance in CoNS from livestock and humans with occupational exposure [12], the potential transmission of multidrug-resistant CoNS from livestock to humans needs to be elucidated. Of particular concern is that the eco-epidemiological context of CoNS is different from *S. aureus*, and these features strongly vary among the different CoNS species, suggesting potential intra-species AMR diversity and dynamics. 

Pig farming is one of the major agrobusinesses in the countries of Europe, America, and some of Southeast Asia. This has intensified concern about the re-emergence and spread of AMR which has implications for human, animal, and environmental health due to the excessive or previous use of antimicrobial agents in pig farms. These could have promoted the selection of AMR in CoNS and the dissemination of critical AMR genes across the pig farm setting. Hence, the present study characterized the mechanisms of AMR and the intra-host species and intra-species AMR diversity of a collection of CoNS previously recovered from the nasal cavities of healthy pigs and pig farmers [13]. Moreover, the frequency of pig-to-pig and pig-to-human (or human-to-pig) transmission levels was determined. 

## 2. Materials and Methods

### 2.1. Bacterial Collection

One-hundred-and-one CoNS strains were previously recovered from the nasal samples of 40 pigs from 4 Spanish pig farms (A–D, 10 pigs/farm) and of 10 farmers (2, 3, 2, and 3 individuals in farms A, B, C, and D, respectively) [13], and they were included in this study for further characterization. The pig herd size, and their age and weight as well as the description of nasal sample processing from the pigs and pig farmers are presented in our previous study [13]. Specifically, farm A had a total of 6000 piglets (average age: 9 weeks); farm B had 15,000 piglets (average age: 4–5 weeks); farm C had 600 piglets (average age: 4–5 weeks); and farm D had 400 piglets (average age: 6 weeks). All the pig farmers worked directly with the pigs.

From these 101 CoNS strains, 75 were considered non-repetitive after their AMR phenotypes and genotypes were determined; they corresponded to one strain of each species per sample or more than one if they presented different AMR phenotypes/genes (Table 1). This collection of 75 non-repetitive CoNS strains was further characterized and considered in this study. The research performed both in the previous and in the present study was reviewed and approved by the ethical research committee of the University of Zaragoza, Spain (ref PI58/21), and by the Ethical Committee of the University of La Rioja. All procedures were carried out following all applicable national, and/or international guidelines for human sample experiments (as described in the revised Helsinki Declaration). Concerning the ethical use of animals, this study adhered to specific directives: 2010/63/EU, Spanish laws 9/2003 and 32/2007, RD 178/2004 and RD 1201/2005.

### 2.2. Antimicrobial Susceptibility Testing and Characterization of Resistance Genes

Antibiotic susceptibility tests for thirteen agents were performed by agar disk diffusion method on all the CoNS strains following the recommendations and breakpoints of the European Committee on Antimicrobial Susceptibility Testing [14]. The antimicrobial agents tested were as follows (µg/disk): penicillin (10), cefoxitin (30), erythromycin (15), clindamycin (2), gentamicin (10), tobramycin (10), tetracycline (30), ciprofloxacin (5), chloramphenicol (30), linezolid (10), mupirocin (200), and trimethoprim–sulfamethoxazole (1.25 + 23.75). The minimum inhibition concentration (MIC) of all strains carrying linezolid resistance genes was tested using bioMérieux Linezolid Etest^®^ strips (Marcy l’Étoile, France), and the results were interpreted following the EUCAST 2022 breakpoints. The CoNS strains that presented resistance to ≥3 classes of the antimicrobial agents tested were considered multidrug-resistant (MDR) [15]. In the case of *S. sciuri*, due to the intrinsic carriage in this species of the *salA* gene (associated with clindamycin resistance), this antibiotic was not considered for MDR categorization. 

The presence of the following resistance genes was tested by PCR, and they were selected according to the antimicrobial resistance phenotype: beta-lactams (*blaZ*, *mecA*, and *mecC*), erythromycin and/or clindamycin (*ermA*, *ermB*, *ermC*, *ermT*, *erm43*, *lnuA*, *lnuB*, *vgaA*, *msrA*, *mphC* and *salA*), tetracycline (*tetK*, *tetL*, *tetM*, and *tetO*), aminoglycosides (*aac6′-aph2″* and *ant4′*), chloramphenicol (*fexA*, *fexB*, *catA*, *cat*_PC194_, *cat*_PC221_, and *cat*_PC223_), linezolid (*cfr*, *cfrD*, *optrA* and *poxtA*), trimethoprim-sulfamethoxazole (*dfrA*, *dfrD*, *dfrG*, and *dfrK*), and mupirocin (*mupA*). 

### 2.3. Molecular Typing of S. epidermidis and MRCoNS Strains

The sequence types of all the *S. epidermidis* strains were determined via MultiLocus Sequence Typing (MLST). The seven housekeeping genes of *S. epidermidis* (*acrC*, *aroE*, *gtr*, *pyrR*, *mutS*, *tpi*, and *yqiL*) were amplified, and the sequence type (ST) was assigned according to the MLST database (https://pubmlst.org/, accessed on 20 March 2023). Moreover, the SCC*mec* typing of all the MRCoNS was performed by multiplex PCRs as previously described [16]. 

### 2.4. Tests for Virulence Genes

The presence of *tst*, *lukS-PV/lukF-PV*, *eta*, and *etb* genes (encoding the toxin of toxic shock syndrome, Panton–Valentine leucocidin, and exfoliative toxins A and B, respectively) were investigated by PCR on every strain. 

Primers and conditions of PCRs for AMR genes, MLST, and virulence factors are included in Supplementary Table S1. Positive controls from the collection of the Universidad de La Rioja were included in all the PCR assays in this study.

### 2.5. Statistical Analysis

AMR data were presented in tables, and a chart on the frequencies of resistance to each type of antimicrobial agent was plotted. The association between the frequencies of resistance to each antibiotic, MDR phenotype, and the individual farms was determined using the Chi-square test, and outcomes with a probability <0.05 were considered statistically significant.

## 3. Results

**Figure 1:**
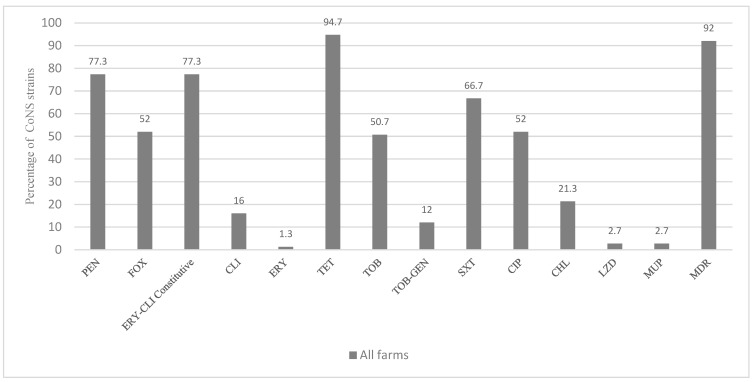
Frequency of antimicrobial resistance in the CoNS strains recovered from nasal cavities of healthy pigs and pig farmers. Abbreviations CHL: chloramphenicol; CLI: clindamycin; CIP: ciprofloxacin; ERY: erythromycin; FOX: cefoxitin; GEN: gentamicin; LZD: linezolid; MUP: mupirocin; MDR: multidrug resistance; PEN: penicillin; SXT: sulfamethoxazole–trimethoprim; TET: tetracycline; TOB: tobramycin.

From our previous study [13], a total of 101 CoNS of nine species were recovered and identified from 72.5% and 60% of pigs and pig farmers tested, respectively (Table 1). From these 101 CoNS, 75 non-repetitive strains were selected after determining their phenotypes/genotypes of AMR. Of the 75 non-repetitive strains (62 from pigs and 13 from pig farmers), 92% showed a multidrug resistance (MDR) phenotype (Table 1 and Figure 1); specifically, 83.6% and 100% of the non-repetitive CoNS from pigs and pig farmers presented an MDR phenotype, respectively (Table 1). All strains were *lukS-PV/lukF-PV-*, *tst-*, *eta-*, and *etb*-negative. 

### 3.1. Antimicrobial Resistance Phenotypes and Genotypes of Non-Repetitive CoNS

**Figure 2:**
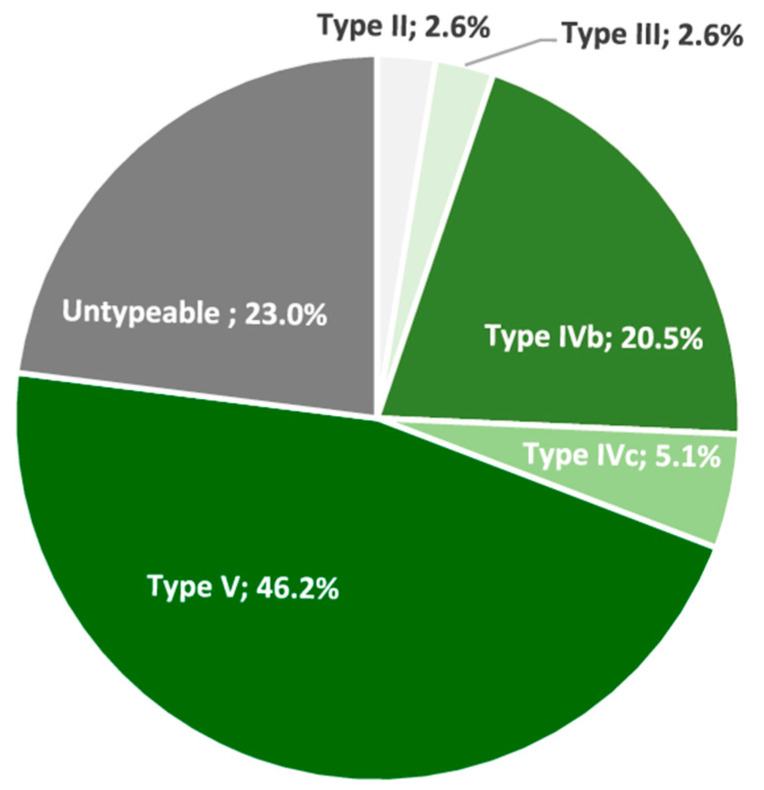
Frequency of the types of SCC*mec* mobile elements identified in the MRCoNS nasal carriers.

All *S. sciuri* strains carried the intrinsic *salA* gene. The following AMR phenotypes were detected among the non-repetitive CoNS (percentage of strains/genes detected): tetracycline (94.7/*tetK*, *tetL*, *tetM*, and *tetO*), penicillin (77.3/*blaZ*), erythromycin–clindamycin-constitutive (77.3/*ermA*, *ermC*, *ermT*, and *erm43*), sulfamethoxazole–trimethoprim (66.7/*dfrA*, *dfrD*, *dfrG*, and *dfrK*), ciprofloxacin (52), tobramycin (50.7/*ant4′*), chloramphenicol (21.3/*fexA* and *cat*_PC221_), clindamycin (16/*lnuA*, *lnuB*, and *salA*), gentamicin–tobramycin (12/*aac6′-aph2″*), linezolid (2.7/*cfr*), mupirocin (2.7/*mupA*), and erythromycin (1.3/*msrA*) (Figure 1, Table 2 and Table 3). About 52% of CoNS were *mecA*-positive (i.e., MRCoNS), and they were associated with SCC*mec* types V (46.2%), IVb (20.5%), and IVc (5.1%). However, 23% of MRCoNS were SCC*mec* non-typeable (Figure 2).

### 3.2. Comparison of AMR Phenotype Frequencies in the Pig Farms

To compare the AMR frequencies of non-repetitive CoNS strains from pigs and pig farmers of the four pig farms (A–D), individual chi-squared tests against every antimicrobial agent were computed. Erythromycin–clindamycin resistance (in all cases of constitutive character) was significantly higher among CoNS strains from pigs and pig farmers in farm A than strains from the other farms (*p* = 0.018) (Table 2). CoNS strains from pigs and pig farmers in Farm B had significantly higher tobramycin and ciprofloxacin resistances than strains from other farms (*p* < 0.05); whereas CoNS strains from pigs and pig farmers in Farm C had the highest resistance to sulfamethoxazole–trimethoprim (*p* = 0.009). For the other antibiotics’ resistances and the MDR phenotype, no significant associations between the farms were detected (*p* > 0.05) (Table 2). 

### 3.3. Unusual Antimicrobial Resistance Genes

Interestingly, the linezolid resistance gene *cfr* was detected in two chloramphenicol-resistant CoNS strains from a pig and a pig farmer (Table 3). One of these strains expressed phenotypic resistance to linezolid (*S. saprophyticus*, MIC: 12 μg/mL), but the other was susceptible to linezolid (*S. epidermidis-*ST16, MIC: 1.5 μg/mL) (Table 3). The *ermT* gene was detected in 14 strains of five CoNS species (*S. chromogenes*, *S. epidermidis*, *S. borealis*, *S. sciuri*, and *S. hyicus*) (Table 3 and Table 4). Moreover, the *erm43* gene was detected in eight CoNS of four different species (*S. epidermidis*, *S. chromogenes*, *S. haemolyticus*, and *S. borealis*), while *mupA* gene was detected in two strains of the species *S. epidermidis* and *S. sciuri* (Table 3 and Table 4). 

### 3.4. Antimicrobial Resistome Diversity across Pigs and Pig Farmers

**Figure 3:**
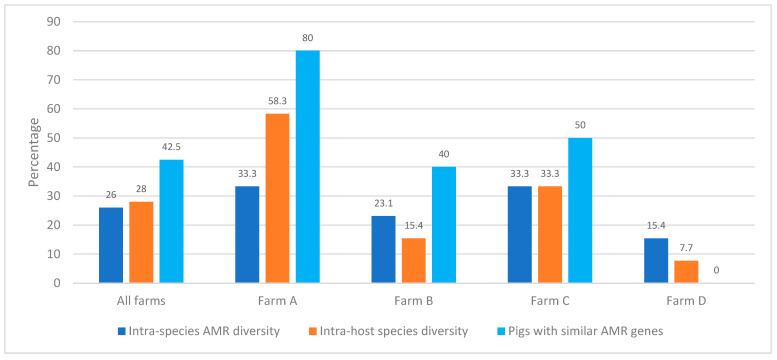
Frequency of intra-species AMR and intra-host species diversities of CoNS among healthy pigs and pig farmers. Note: The number of individuals included 10 pigs from each farm (a total of 40 pigs) and 10 workers from the four pig farms.

A total of 28% of the pigs and pig farmers had intra-host species diversity (>1 CoNS species in a host), while 26% had intra-species AMR diversity (same species with >1 AMR profile) (Figure 3 and Table 3). Pig-to-pig nasal transmission of CoNS with similar MDR genes and SCC*mec* types was detected in 35% of pigs (Figure 3 and Table 3). In farm A, *S. sciuri* strains carrying the same resistome and SCC*mec* type were found in pigs 2, 3, and 9; *S. borealis* in pigs 2, 7, 8, and 9; *S. chromogenes* in pigs 3, 7, 8, and 10; *S. epidermidis*-ST25 in pigs 2 and 7; *S. hyicus* in pigs 1 and 6; and *S. pasteuri* in pigs 8 and 10 (Table 3). In farm B, similar *S. hycius* strains were found in pigs 2 and 3; and *S. borealis* was found in pigs 4 and 5 (Table 3); whereas in farm C, similar *S. sciuri* strains were found in pigs 3, 7, 8, and 10; and *S. xylosus* in pigs 9 and 10 (Table 3 and Table 4). No similar strains were detected in farm D. 

## 4. Discussion

There is a worry about the potential of AMR to assume pandemic status. Consequently, studies have been intensified to understand the molecular ecology and transmission of the resistomes in bacteria that have the potential for zoonoses, such as *Staphylococcus*. Some previous studies have reported the detection and AMR phenotypes of nasal CoNS from healthy pigs and pig farmers [11,12,17,18,19,20,21,22,23,24,25]. However, we are not aware of any that investigated the diversity level of AMR in CoNS across pigs, pigs-to-pig farmers, and pig farmers-to-pigs, especially in Spain. The AMR profiles detected in our study greatly varied, with high levels of resistance to tetracycline, chloramphenicol, and erythromycin. The high level of tetracycline resistance mostly mediated by *tetM* and *tetL* genes, could be associated with the high use of this agent in animal husbandry [17]. Florfenicol is also frequently used in livestock, which could contribute to the persistence of chloramphenicol resistance and the emergence of linezolid cross-resistance [26]. In this sense, is of concern the detection of linezolid resistance genes in two strains. Linezolid has never been licensed for use in livestock [27]. However, other classes of antibiotics (i.e., phenicols, lincosamides, pleuromutilins, and streptogramin A) could have contributed to the increased risks for cross-resistance to linezolid through the *cfr* gene [28,29]. 

Aside from the *cfr*-carrying *S. saprophyicus* and *S. epidermidis*, several MDR*-S. borealis* strains carrying SCC*mec* type-V were detected among pigs from three of the four farms studied. To our knowledge, this is the first report on the molecular characterization of AMR genes of MDR-*S. borealis* strains from healthy pigs in the literature. The *S. borealis* was first described by whole genome sequencing and ascribed to a distinct species due to the significant phylogenetic distance from *S. haemolyticus* [7]. Despite being a relatively new species previously detected in strains from human skin and blood samples, it needs to be monitored and fully characterized to determine its potential to spread MDR and critical AMR genes in other ecological niches. The presence of *cfr* gene did not translate to phenotypic LZD resistance on both the disc diffusion test and E-test in the *S. epidermidis* strain. These results confirm the silent emergence of LZD resistance at the molecular level in *S. epidermidis* from a pig. It appears that the pig farm environment favours the persistence of linezolid resistance and MDR genes [27,30]. 

Many of the identified AMR genes in the CoNS strains are commonly found within mobile genetic elements, such as *mecA*. In this sense, the MRSA strains have long been considered to have originated from the acquisition of SCC*mec* from MRCoNS. However, whether the same SCC*mec* types are present in MRSA and MRCoNS that reside in the same nasal niche needs to be elucidated. Even though the high-level AMR genes detected were from CoNS strains (often considered harmless), they can exchange mobile genetic elements with pathogenic species [31]. Unfortunately, the molecular surveillance of these multiresistant CoNS is underrated [31]. It is important to acknowledge the frequent detection of *S. epidermidis* ST59, a clone that has very high community transmission potential [32] and which may facilitate the transmission and persistence of AMR genes in various ecological niches. One of the *cfr-*carrying strains is an *S. epidermidis*-ST16: this genetic lineage has previously been reported to cause bloodstream infection, but in their study, the strain case did not carry the *cfr* gene [33], as detected in the present study. 

The results obtained with the statistical analysis performed indicate that different factors in pig farming could be involved in some AMR rates detected among CoNS, as in the case of the significantly high rates of ciprofloxacin and chloramphenicol in farm A compared to others. This difference could be due to the hygienic status of the farm, the population of herds [34], and other potential factors that need to be thoroughly investigated.

Some strains identified in this study had phenotypic resistance (especially to penicillin) but did not harbour the corresponding genes tested. Perhaps, this could be due to certain amino acid changes or polymorphisms in the *blaZ* gene, or perhaps the *mecA* gene in the bacteria mediated the penicillin resistance without expression of *blaZ* gene [35].

It is worth mentioning the detection of the *ermT* gene in some species of CoNS causes erythromycin–clindamycin constitutive resistance, which is an unusual mechanism in CoNS. To our knowledge, this study is the first to report the presence of this gene in CoNS strains of pigs and pig farmers in Spain. Although the gene has previously been reported in an *S. haemolyticus* strain [27] in an environmental sample from a pig farm, there is a paucity of data on the description of the *ermT* gene in CoNS species. This pattern of phenotypic resistance expressed by the *ermT* gene in our CoNS strains is quite different to the typical erythromycin–clindamycin-inducible resistance phenotype it confers in the methicillin susceptible*-S. aureus* of the CC398 lineage [36]. Perhaps there is a silent evolution of this gene in non-*aureus* staphylococci, which deserves to be studied in detail. 

Another point to mention is the detection of similar species of CoNS with different AMR profiles and genes in the same host. This underscores the enormous challenge these strains could pose in the control of AMR at the farm level. More specifically, as some of the CoNS strains carrying similar AMR profiles were identified in ≥3 pigs on the same farm, this is a strong indicator of transmission events of similar CoNS across the pig herds. 

This study is not without limitations. Using whole genome sequencing could be useful to detect the single nucleotide polymorphism difference between strains with similar AMR profiles and lend better credence to confirming the transmission of the CoNS strains between pigs and even across pigs and pig farmers. Moreover, analyses of repeat samples from the hosts with similar AMR profiles, SCC*mec* type, and genetic lineages could be of value to confirm transmission events. 

## 5. Conclusions

The high level of MDR, intra-host species, and intra-species AMR diversity in the nasal CoNS strains from healthy pigs highlights their ability to be long-term AMR reservoirs and vectors of transmission to pig farmers. As the incidence of MLS_b_, tobramycin, ciprofloxacin, and sulfamethoxazole–trimethoprim resistances significantly vary by farms, specific control measures should be taken, as is the control in antibiotic use in the farms. Moreover, it has been demonstrated that various CoNS species from healthy pigs and pig farmers carried AMR genes conferring resistance to clinically relevant antibiotics. In addition, the detection of MDR-*S. borealis* and *cfr*-carrying strains require comprehensive and continuous surveillance of CoNS at pig farm levels. The selective inclusion of chloramphenicol resistance as a marker for linezolid resistance could facilitate its early detection. 

## Figures and Tables

**Table 1:** Coagulase-negative staphylococci from healthy pigs and pig farmers and those with MDR phenotype from the four farms (A–D).

CoNS Species	Total Strains	Non-Repetitive Strains ^a^												
		Strains with MDR Phenotype ^b^			Strains with MDR Phenotype in Pigs					Strains with MDR Phenotype in Pig Farmers				
		Pigs	Pig Farmers	Pigs and Pig farmers	All Farms	Farm A	Farm B	Farm C	Farm D	All Farms	Farm A	Farm B	Farm C	Farm D
*S. sciuri*	29	17	0	17	17	4	0	13	0	0	0	0	0	0
*S. haemolyticus*	5	3	1	4	3	0	3	0	0	1	0	0	0	1
*S. borealis*	12	10	0	10	10	5	4	0	1	0	0	0	0	0
*S. chromogenes*	15	11	2	9	7	5	1	0	1	2	0	0	0	2
*S. epidermidis*	13	5	5	10	5	4	1	0	0	5	2	2	1	0
*S. hyicus*	11	8	1	9	8	3	3	2	0	1	0	1	0	0
*S. saprophyticus*	7	3	1	4	3	2	1	0	0	1	0	1	0	0
*S. simulans*	4	1	3	4	1	1	0	0	0	3	0	0	1	2
*S. xylosus*	3	2	0	0	0	0	0	0	0	0	0	0	0	0
*S. pasteuri*	2	2	0	2	2	2	0	0	0	0	0	0	0	0
Total (%)	101	62	13	69 (92)	56 (83.6)	26	13	15	2	13 (100)	2	4	2	5

^a^ Non-repetitive strains: one of each species per sample, or more than one if they presented a different AMR phenotype. ^b^ MDR: resistance to at least 3 families of antibiotics. In *S. sciuri*, clindamycin resistance was not considered for MDR analyses (this species has an intrinsic mechanism of lincomycin resistance).

**Table 2:** Comparison of the frequencies of antimicrobial resistance phenotypes among CoNS strains from healthy pigs and pig farmers in farms A to D.

Antimicrobial Resistance Phenotype	Farm A (%)	Farm B (%)	Farm C (%)	Farm D (%)	*χ* ^2^	*p* Value
PEN	23 (76.7)	15 (88.2)	17 (80.9)	3 (42.9)	5.078	0.166
FOX	16 (53.3)	10 (58.8)	10 (47.6)	3 (42.9)	0.734	0.865
ERY-CLI constitutive	28 (93.3)	12 (40)	12 (57.1)	6 (85.7)	9.987	0.018 *
CLI	0	4 (13.3)	7 (33.3)	1 (14.3)	11.141	0.011 *
ERY	1 (3.3)	0	0	0	1.520	0.677
TET	30 (100)	15 (88.2)	20 (95.2)	6 (85.7)	4.208	0.239
TOB	17 (56.7)	14 (82.3)	5 (23.8)	2 (28.6)	14.688	0.002*
TOB-GEN	2 (6.7)	2 (11.7)	3 (14.3)	2 (28.6)	2.733	0.434
SXT	24 (80)	12 (70.6)	8 (38.1)	6 (85.7)	11.375	0.009 *
CIP	12 (40)	15 (88.2)	9 (42.9)	3 (42.9)	11.611	0.008 *
CHL	9 (30)	4 (23.5)	2 (9.5)	1 (14.3)	3.344	0.341
LZD	1 (3.3)	1 (5.9)	0	0	1.496	0.683
MUP	0	0	2 (9.5)	0	5.284	0.152
MDR	28 (93.3)	17 (100)	17 (80.9)	7 (100)	5.642	0.130

The number of CoNS strains from the farms were as follows: Farm A = 30, Farm B = 17, Farm C = 21, and Farm D = 7; * Significant association determined via two-tailed chi-squared test at 95% confidence interval (CI). Abbreviations: CHL: chloramphenicol; CLI: clindamycin; CIP: ciprofloxacin; ERY: erythromycin; FOX: cefoxitin; GEN: gentamicin; LZD: linezolid; MUP: mupirocin; MDR: multidrug resistance; PEN: penicillin; SXT: sulfamethoxazole–trimethoprim; TET: tetracycline; TOB: tobramycin.

**Table 3:** Intra-host species and intra-species AMR diversity of coagulase-negative staphylococci from healthy pigs and pig farmers.

Host/Farm	Staphylococcal Species	AMR Phenotype	AMR Genes Detected	LZD Resistance		ST	SCC*mec* Type
				Genes	MIC ^a^		
Pig 1/A	*S. epidermidis*	PEN-FOX-TET-ERY-CLI-SXT-TOB	*blaZ*, *mecA*, *tetL*, *tetM*, *ermB*, *dfrD*, *aac6′-aph2″*, *ant4′*	-	-	ST25	IVc
	*S. hyicus*	PEN-TET-ERY- CLI	*blaZ*, *tetL*, *ermC*	-	-	-	-
	*S. simulans*	TET-ERY-CLI-SXT-GEN	*tetK*, *ermA*, *dfrG*, *aac6′-aph6″*	-	-	-	-
Pig 2/A	*S. sciuri*	PEN-FOX-TET-ERY-CLI-SXT-CHL-CN-TOB-CIP	*mecA*, *tetL*, *tetM*, *ermA*, *ermB*, *ermC*, *lnuA*, *salA*, *dfrD*, *fexA*, *aac6′-aph2″*, *ant4′*	ND	-	-	IVb
	*S. epidermidis*	PEN-FOX-TET-ERY-CLI-SXT-TOB	*blaZ*, *mecA*, *tetM*, *erm43*, *ermC*, *dfrG*, *dfrK*, *ant4′*	-	-	ST25	V
	*S. borealis*	PEN-FOX-TET-ERY-CLI-SXT-CHL-TOB-CIP	*blaZ*, *mecA*, *tetL*, *tetM*, *ermC*, *ermT*, *lnuB*, *dfrK*, *cat*_PC221_, *fexA*, *ant4′*	ND	-	-	V
Pig 3/A	*S. sciuri*	PEN-FOX-TET-ERY-CLI-SXT-CHL-TOB-CIP	*blaZ*, *mecA*, *tetL*, *tetM*, *ermC*, *ermT*, *lnuB*, *dfrK*, *cat*_PC221_, *fexA*, *ant4′*	ND	-	-	IVb
	*S. sciuri*	PEN-FOX-TET-ERY-CLI-SXT-CHL-TOB-CIP	*mecA*, *tetL*, *tetM*, *ermC*, *msrA*, *dfrK*, *cat*_PC221_, *ant4′*	ND	-	-	IVb
	*S. chromogenes*	PEN-TET-ERY-CLI-TOB-SXT	*blaZ*, *tetL*, *erm43*, *ermT*, *dfrA*, *dfrG*, *dfrK*, *ant4′*	-	-	-	-
Pig 4/A	*S. chromogenes*	TET-ERY-CLI-SXT-TOB	*tetL*, *tetM*, *ermA*, *dfrA*, *ant4′*	-	-	-	-
	*S. chromogenes*	PEN-FOX-TET-ERY-CLI-SXT	*blaZ*, *mecA*, *tetL*, *erm43*, *ermA*, *ermT*, *dfrA*, *dfrG*, *dfrK*	-	-	-	NT
Pig 7/A	*S. chromogenes*	PEN-TET-ERY-CLI-SXT-TOB	*blaZ*, *tetL*, *erm43*, *ermT*, *dfrA*, *dfrG*, *dfrK*, *ant4′*	-	-	-	-
	*S. epidermidis*	PEN-FOX-TET-ERY-CLI-SXT-TOB	*blaZ*, *mecA*, *tetM*, *ermC*, *dfrK*, *aac6′-aph2″*, *ant4′*	-	-	ST25	IVc
	*S. saprophyticus*	PEN-FOX-TET-ERY-CLI-SXT	*blaZ*, *mecA*, *tetL*, *tetM*, *ermC*, *ermA*, *dfrK*	-	-	-	III
	*S. borealis*	PEN-FOX-TET-ERY-CLI-SXT-CHL-TOB-CIP	*blaZ*, *mecA*, *tetL*, *tetM*, *ermC*, *ermT*, *lnuB*, *cat*_PC221_, *fexA*, *dfrK*, *ant4′*	ND	-		V
Pig 8/A	*S. chromogenes*	TET-ERY-CLI-TOB	*tetL*, *tetM*, *ermC*, *ant4′*	-	-	-	-
	*S. chromogenes*	TET-ERY-CLI	*tetL*, *ermC*	-	-	-	-
	*S. epidermidis*	TET-ERY-CLI-SXT	*blaZ*, *tetO*, *tetL*, *tetM*, *ermC*, *dfrK*	-	-	ST977	-
	*S. borealis*	PEN-FOX-TET-ERY-CLI-TOB-CIP	*blaZ*, *mecA*, *tetL*, *tetM*, *erm43*, *dfrA*, *dfrG*, *dfrK*, *ant4′*	-	-	-	V
	*S. borealis*	PEN-FOX-TET-ERY-CLI-SXT-CHL-TOB-CIP	*blaZ*, *mecA*, *tetL*, *tetM*, *ermC*, *ermT*, *lnuB*, *dfrK*, *fexA*, *ant4′*	ND	-	-	V
	*S. pastueri*	PEN-FOX-TET-ERY-CLI-SXT- TOB-CIP	*blaZ*, *mecA*, *tetK*, *tetL tetM*, *ermC*, *dfrK*, *ant4′*	-	-	-	V
Pig 9/A	*S. sciuri*	PEN-TET-ERY-CLI-SXT-CHL-TOB	*mecA*, *tetL*, *tetM*, *ermC*, *lnuA*, *fexA*, *dfrK*, *ant4′*, *aac6′-aph2″*	ND	-	-	IVb
	*S. borealis*	PEN-FOX-TET-ERY-CLI-SXT-CHL-TOB-CIP	*blaZ*, *mecA*, *tetL*, *tetM*, *ermC*, *ermT*, *lnuB*, *cat*_PC221_, *fexA*, *dfrK*, *ant4′*	ND	-	-	V
Pig 10/A	*S. chromogenes*	TET-ERY-CLI	*tetL*, *ermC*	-	-	-	-
	*S. saprophyticus*	FOX-TET-ERY-CHL-CLI-TOB-SXT	*mecA*, *tetL*, *tetM*, *ermC*, *dfrK*, *fexA*, *ant4′*	*cfr*	12	-	V
	*S. pasteuri*	PEN-FOX-TET-ERY-CLI-SXT-TOB-CIP	*blaZ*, *mecA*, *tetL*, *tetM*, *ermC*, *dfrG*, *dfrK*, *ant4′*	-	-	-	V
Farmer 1/A	*S. epidermidis*	PEN-FOX-TET-ERY- SXT-CIP	*blaZ*, *mecA*, *tetO*, *msrA*, *dfrA*, *dfrG*	-	-	ST59	V
	*S. epidermidis*	PEN-FOX-TET- SXT-CIP	*blaZ*, *mecA*, *tetL*, *dfrA*, *dfrG*	-	-	ST59	V
Pig 1/B	*S. haemolyticus*	PEN-TET-ERY-CLI-SXT-GEN-TOB	*blaZ*, *mecA*, *tetL*, *tetM*, *erm43*, *ermC*, *dfrA*, *aac6′-aph2″*, *ant4′*	-	-	-	V
	*S. haemolyticus*	PEN-FOX-TET-ERY-CLI-GEN-TOB-CIP	*mecA*, *tetL*, *ermA*, *ermT*, *dfrA*, *dfrG*, *aac6′-aph2″*, *ant4′*	-	-	-	V
	*S. epidermidis*	PEN-TET-ERY-CLI-TOB	*blaZ*, *tetL*, *tetM*, *tetK*, *ermC*, *ant4′*	-	-	ST100	-
	*S. hyicus*	PEN-TET-ERY-CLI-TOB-GEN-CIP	*blaZ*, *tetL*, *ermT*, *aac6′-aph2″*	-	-	-	-
Pig 4/B	*S. borealis*	PEN-FOX-TET-ERY-CLI-SXT-TOB-CIP	*mecA*, *tetK*, *tetL*, *ermA*, *ermC*, *dfrK*, *ant4′*	-	-	-	V
	*S. borealis*	PEN-FOX-TET-ERY-CLI-CHL-SXT-GEN-TOB-CIP	*blaZ*, *mecA*, *tetL*, *tetM*, *ermT*, *fexA*, *dfrK*, *aac6′-aph2″*, *ant4′*	ND	-	-	V
	*S. haemolyticus*	PEN-TET-CLI-GEN-TOB-CIP	*tetL ermC*, *lnuA*, *aac6′-aph2″*, *ant4′*	-	-	-	-
Pig 5/B	*S. borealis*	PEN-FOX-TET-ERY-CLI-CHL-SXT-GEN-TOB-CIP	*blaZ*, *mecA*, *tetL*, *tetM*, *ermA*, *ermT*, *cat*_PC221_, *fexA*, *dfrK*, *aac6′-aph2″*, *ant4′*	ND	-	-	V
	*S. borealis*	PEN-FOX-TET-ERY-CLI-SXT-TOB-CIP	*mecA*, *tetK*, *tetL*, *ermA*, *ermC*, *dfrK*, *ant4′*	-	-	-	V
Farmer 1/B	*S. epidermidis*	PEN-FOX-TET-CLI-CHL-SXT-TOB-CIP	*blaZ*, *mecA*, *tetL*, *tetK*, *fexA*, *dfrK*, *ant4′*	*cfr*	1.5	ST16	V
	*S. hyicus*	PEN-FOX-TET-CIP-SXT	*blaZ*, *mecA*, *tetK*, *tetO*, *dfrA*, *dfrG*	-	-	-	NT
	*S. saprophyticus*	PEN-FOX-TET-ERY-CLI-SXT-TOB-GEN-SXT-CIP	*blaZ*, *mecA*, *tetK*, *tetM*, *ermC*, *dfrG*, *ant4′*, *aac6′-aph2″*	-	-	-	V
Pig 1/C	*S. sciuri*	PEN-FOX-TET-ERY-CLI-SXT-CIP	*mecA*, *tetL*, *tetM*, *ermB*, *erm43*, *dfrK*	-	-	-	NT
	*S. chromogenes*	TET-ERY-CLI	*tetM*, *ermC*, *lnuB*	-	-	-	-
	*S. hyicus*	PEN-TET-ERY-CLI-SXT-CIP	*blaZ*, *tetL*, *ermT*, *dfrK*	-	-	-	-
Pig 4/C	*S. sciuri*	PEN-FOX-TET-ERY-CLI-TOB	*mecA*, *tetL*, *tetM*, *ermB*, *dfrK*, *ant4′*	-	-	-	NT
	*S. sciuri*	PEN-FOX-TET-ERY-CLI-CIP-TOB-GEN	*mecA*, *tetL*, *ermC*, *aac6′-aph2″*	-	-	-	V
Pig 6/C	*S. sciuri*	PEN-TET-ERY-CLI-SXT-CIP	*mecA*, *tetL*, *tetM*, *ermT*, *dfrG*, *dfrK*	-	-	-	NT
	*S. sciuri*	PEN-FOX-TET-CLI-TOB	*mecA*, *tetL*, *tetM*, *lnuA*, *ant4′*	-	-	-	IVb
Pig 8/C	*S. sciuri*	TET-CLI-PEN-TOB	*tetL*, *lnuA*, *ant4′*	-	-	-	-
	*S. sciuri*	PEN-FOX-TET-ERY-CLI	*mecA*, *tetM*, *ermB*	-	-	-	IVb
	*S. sciuri*	PEN-FOX-TET-ERY-CLI-SXT	*mecA*, *tetL*, *tetM*, *ermB*, *dfrK*	-	-	-	-
Pig 9/C	*S. hyicus*	PEN- FOX-TET-CLI-SXT-TOB-GEN-CIP	*blaZ*, *mecA*, *tetM*, *lnuA*, *lnuB*, *dfrD*, *aac6′-aph2″*	-	-	-	V
	*S. xylosus*	PEN-TET	*blaZ*, *tetK*	-	-	-	-
Pig 10/C	*S. sciuri*	PEN-FOX-TET-ERY-CLI-SXT-CIP	*mecA*, *tetL*, *tetM*, *ermB*, *dfrK*	-	-	-	NT
	*S. sciuri*	TET-ERY-CLI-CHL-SXT-CIP	*tetL*, *tetM*, *ermA*, *lnuA*, *cat*_PC221_, *dfrK*	ND	-	-	-
	*S. xylosus*	PEN-TET	*blaZ*, *tetK*	-	-	-	-
Farmer 1/C	*S. epidermidis*	PEN-TET-ERY-CLI-TOB-MUP	*blaZ*, *tetK*, *tetL*, *tetM*, *erm43*, *dfrA*, *dfrK*, *ant4′*, *mupA*	-	-	ST100	-
	*S. simulans*	TET-CLI-CHL	*tetK*, *lnuA*, *fexA*	ND	-	-	-
Farmer 2/D	*S. simulans*	PEN-FOX-TET-ERY-CLI-TOB-GEN	*blaZ*, *mecA*, *tetL*, *ermA*, *aac6′-aph2″*	-	-	-	NT
	*S. simulans*	TET-ERY-CLI-SXT	*tetM*, *ermC*, *dfrG*	-	-	-	-
	*S. haemolyticus*	PEN-FOX-TET-CLI-SXT-TOB-GEN-CIP	*blaZ*, *mecA*, *tetK*, *lnuA*, *dfrG*, *aac6′-aph2″*	-	-	-	II
Farmer 3/D	*S. chromogenes*	TET-ERY-CLI-SXT	*tetL ermT*, *dfrA*, *dfrG*	-	-	-	-
	*S. chromogenes*	ERY-CLI-CHL-SXT	*mecA*, *tetL*, *tetM*, *ermC*, *dfrK*, *fexA*	ND	-	-	IVb

Abbreviations: CHL: chloramphenicol; CLI: clindamycin; CIP: ciprofloxacin; ERY: erythromycin; FOX: cefoxitin; GEN: gentamicin; LZD: linezolid; MUP: mupirocin; PEN: penicillin; SXT: sulfamethoxazole–trimethoprim; TET: tetracycline; TOB: tobramycin. ST: Sequence type; NT: Non-typeable; -: Not tested; ND: Not detected. *Note:* All strains were *lukS-PV/lukF-PV*-, *tst-, eta*-, and *etb*-negative; ^a^ Linezolid MIC (μg/mL) was tested in the strains that carried linezolid resistance genes.

**Table 4:** CoNS with single antimicrobial resistance profile from healthy pigs and pig farmers.

Host/Farm	Staphylococcal Species	AMR Phenotype	AMR Genes Detected	ST	SCC*mec* Type
Pig 5/A	*S. hyicus*	PEN-TET-ERY-CLI-SXT	*blaZ*, *tetM*, *ermC*, *dfrA*, *dfrG*	-	-
Pig 6/A	*S. hyicus*	PEN-TET-ERY-CLI	*blaZ*, *tetL*, *ermC*	-	-
Pig 2/B	*S. hycius*	CLI-SXT-GEN-TOB-CIP	*lnuA*, *lnuB*, *dfrK*, *aac6′-aph2″*, *ant4′*	-	-
Pig 3/B	*S. hyicus*	CLI-SXT-GEN-TOB-CIP	*lnuA*, *lnuB*, *dfrK*, *aac6′-aph2″*, *ant4′*	-	-
Pig 6/B	*S. saprophyticus*	PEN-FOX-TET-ERY-CLI-SXT-TOB-GEN- SXT-CIP	*blaZ*, *mecA*, *tetM*, *ermC*, *dfrA*, *dfrG*, *ant4′*, *aac6′-aph2″*	-	V
Pig 9/B	*S. chromogenes*	PEN-TET-ERY-CLI-GEN-TOB-CIP	*blaZ*, *tetL*, *ermT*, *aac6′-aph2″*, *ant4′*	-	-
Farmer 2/B	*S. epidermidis*	PEN-FOX-TET-ERY-CLI-CHL-SXT-TOB- GEN-CIP	*blaZ*, *mecA*, *tetL*, *tetM*, *ermT*, *lnuB*, *cat*_PC221_, *fexA*, *dfrA*, *dfrK*, *aac6′-aph2″*, *ant4′*	ST59	V
Pig 2/C	*S. sciuri*	PEN-FOX-TET-CLI-CIP-TOB-GEN-MUP	*mecA*, *tetL*, *tetM*, *lnuA*, *ant4′*, *mupA*	-	IVb
Pig 3/C	*S. sciuri*	PEN-FOX-TET-ERY-CLI-SXT-CIP	*mecA*, *tetL*, *tetM*, *ermB*, *dfrK*	-	NT
Pig 5/C	*S. chromogenes*	CLI	*lnuB*	-	-
Pig 7/C	*S. sciuri*	TET-CLI-PEN-TOB	*tetL*, *lnuA*, *ant4′*	-	-
Pig 1/D	*S. chromogenes*	TET-ERY-CLI-SXT-TOB-CIP	*tetL*, *tetM*, *tetK*, *ermC*, *dfrK*, *ant4′*	-	-
Pig 5/D	*S. borealis*	PEN-FOX-TET-ERY-CLI-SXT-TOB-CIP	*blaZ*, *mecA*, *tetL*, *ermT*, *dfrA*, *dfrK*, *ant4′*, *aac6′-aph2″*	-	NT

CLO: chloramphenicol; CLI: clindamycin; CIP: ciprofloxacin; ERY: erythromycin; FOX: cefoxitin; GEN: gentamicin; LZD: linezolid; MUP: mupirocin; PEN: penicillin; SXT: sulfamethoxazole–trimethoprim; TET: tetracycline; TOB: tobramycin; ST: Sequence type; NT: Non-typeable; -: Not tested. *Note:* All strains were *lukS-PV/lukF-PV*-, *tst*-*, eta*-, and *etb*-negative.

## Data Availability

The data generated from this study has been fully presented in the manuscript. However, further requests can be made through the corresponding author.

## Supplementary Materials

The following supporting information can be downloaded at https://www.mdpi.com/2079-6382/12/10/1505/s1:, Table S1. Gene and primer sequences utilized for all PCRs in this study. References [37,38,39,40,41,42,43,44,45,46,47,48,49,50,51,52,53,54,55,56,57,58,59,60] are only cited in the supplementary materials.
