# Supplementary material for: Within-Host Diversity of Coagulase-Negative Staphylococci Resistome from Healthy Pigs and Pig Farmers, with the Detection of cfr-Carrying Strains and MDR-S. borealis
Source: Antibiotics (Basel). 2023 Oct 2;12(10):1505. doi: 10.3390/antibiotics12101505 (PMC10604674; doi:10.3390/antibiotics12101505)
Supplement: Supplementary file 1 [file antibiotics-12-01505-s001.zip › antibiotics-2628966-supplementary.pdf]

Table S1. Genes and primers' sequences utilized for all PCRs in this study.

Gene	Primers' Oligonucleotide (5'⇒3')	Amplicon Size	Reference
	**Antimicrobial Resistance (AMR) Genes**		
blaZ	F: CAGTTCACATGCCAAAGAG<br>R: TACACTCTTGGCGGTTTC	772 bp	[37]
mecA	F: GGGATCATAGCGTCATTATTC<br>R: AACGATTGTGACACGATAGCC	527 bp	[38]
mecC	F: GCTCCTAATGCTAATGCA<br>R: TAAGCAATAATGACTACC	304 bp	[39]
ermA	F: TCTAAAAAGCATGTAAAAGAA<br>R: CTTCGATAGTTTATTAATATTAG	645 bp	[40]
ermB	F: GAAAAGTACTCAACCAAATA<br>R: AGTAACGGTACTTAAATTGTTTA	639 bp	[40]
ermC	F: TCAAAACATAATATAGATAAA<br>R: GCTAATATTGTTTAAATCGTCAAT	642 bp	[40]
ermT	F: CCGCCATTGAAATAGATCCT<br>R: TTCTGTAGCTGTGCTTTCAAAAA	200 bp	[41]
erm43	F: TACAGCAGATGATAACATTG<br>R: GTTGTTTCGATATTTTATTTAAG	609 pb	[42]
mphC	F: ATGACTCGACATAATGAAAT<br>R: CTACTCTTTCATACCTAACTC	900 bp	[37]
msrA	F: GCAAATGGTGTAGGTAAGACAACT<br>R: ATCATGTGATGTAAACAAAAT	399 bp	[43]
lnuA	F: GGTGGCTGGGGGGTAGATGTATTAACTGG<br>R: GCTTCTTTTGAAATACATGGTATTTTTCGATC	323 bp	[44]
lnuB	F: CCTACCTATTGTTTGTGGAA<br>R: ATAACGTTACTCTCCTATTC	499 bp	[45]
salA	F: CTATTAATCGATGAACCAACAAACC<br>R: TTGATTTACCTGTACCATTTCTGC	610 pb	[46]
vgaA	F: AGTGGTGGTGAAGTAACACG<br>R: GGTTCAATACTCAATCGACTGAG	1264 pb	[47]
aac6'-aph2''	F: CCAAGAGCAATAAGGGCATA<br>R: CACTATCATAACCACTACCG	220 bp	[48]
ant4'	F: GCAAGGACCGACAACATTTC<br>R: TGGCACAGATGGTCATAACC	165 bp	[48]
tet(L)	F: CATTTGGTCTTATTGGATCG<br>R: ATTACACTTCCGATTTCGG	456 bp	[49]
tet(M)	F: GTTAAATAGTGTTCTTGGAG<br>R: CTAAGATATGGCTCTAACAA	576 bp	[49]
tet(K)	F: TTAGGTGAAGGGTTAGGTCC<br>R: GCAAACTCATTCCAGAAGCA	697 bp	[49]

tet(O)	F: GATGGCATACAGGCACAGAC<br>R: CAATATCACCAGAGCAGGCT	615 bp	[49]
dfrA	F: CCTTGGCACTTACCAAATG<br>R: CTGAAGATTCGACTTCCC	374 bp	[37]
dfrD	F: TTCTTTAATTGTTGCGATGG<br>R: TTAACGAATTCTCTCATATATATG	582 bp	[37]
dfrG	F: TCGGAAGAGCCTTACCTGACAGAA<br>R: CCCTTTTTGGGCAAATACCTCATTCCA	323 bp	[41]
dfrK	F: GAGAATCCCAGAGGATTGGG<br>R: CAAGAAGCTTTTCGCTCATAAA	423 bp	[41]
cat_{pC221}	F: ATTTATGCAATTATGGAAGTTG<br>R: TGAAGCATGGTAACCATCAC	434 bp	[37]
cat_{pC223}	F: GAATCAAATGCTAGTTTTAACTC<br>R: ACATGGTAACCATCACATAC	283 bp	[37]
cat_{pC194}	F: CGACTTTTAGTATAACCACAGA<br>R: GCCAGTCATTAGGCCTAT	570 bp	[37]
catA	F: GGATATGAAATTTATCCCTC<br>R: CAATCATCTACCCTATGAAT	505 bp	[49]
fexA	F: GTACTTGTAGGTGCAATTACGGCTGA<br>R: CGCATCTGAGTAGGACATAGCGTC	1272 bp	[50]
fexB	F: TTCCCACTATTGGTGAAAGGAT<br>R: GCAATTCCCTTTTATGGACGTT	816 bp	[51]
cfr	F: TGAAGTATAAAGCAGGTTGGGAGTCA<br>R: ACCATATAATTGACCACAAGCAGC	746 bp	[52]
cfrB	F: TGAGCATATACGAGTAACCTCAAGA<br>R: CGCAAGCAGCGTCTATATCA	293 bp	[53]
cfrD	F: AGAAGTCGCAACAAGTGAGGA<br>R: GCAACTGCATGAGTCAAAGAA	595 bp	[54]
optrA	F: AGGTGGTCAGCGAACTAA<br>R: ATCAACTGTTCCCATTCA	1395 bp	[55]
poxtA	F: TCAATGCAGAGCAGGAAGCA<br>R: GGTGGATTTACCGACACCGT	791 bp	[54]
mupA	F: CCCATGGCTTACCAGTTGA<br>R: CCATGGAGCACTATCCGAA	419 pb	[56]
	***S. epidermidis* housekeeping alleles for MLST**		
arcC	F: TGTGATGAGCACGCTACCGTTAG<br>R: TCCAAGTAAACCCATCGGTCTG	465 pb	[57]
aroE	F: CATTGGATTACCTCTTTGTTCAGC<br>R: CAAGCGAAATCTGTTGGGG	430 pb	[57]
gti	F: CAGCCAATTCTTTTATGACTTTT<br>R: GTGATTAAAGGTATTGATTTGAAT	438 pb	[57]
mutS	F: GATATAAGAATAAGGGTTGTGAA<br>R: GTAATCGTCTCAGTTATCATGTT	412 bp	[57]

pyrR	F: GTTACTAATACTTTTGCTGTGTTT<br>R: GTAGAATGTAAAGAGACTAAAATGAA	428 bp	[57]
tpi	F: ATCCAATTAGACGCTTTAGTAAC<br>R: TTAATGATGCGCCACCTACA	424 bp	[57]
yqiL	F: CACGCATAGTATTAGCTGAAG<br>R: CTAATGCCTTCATCTTGAGAAATAA	416 bp	[57]
	**SCC*mec* types**		
SCC*mec* type I	F: GCTTTAAAGAGTGTCGTTACAGG<br>R: GTTCTCTCATAGTATGACGTCC	613 bp	[16]
SCC*mec* type II	F: CGTTGAAGATGATGAAGCG<br>R: CGAAATCAATGGTTAATGGACC	398 bp	[16]
SCC*mec* type III	F: CCATATTGTGTACGATGCG<br>R: CCTTAGTTGTCGTAACAGATCG	280 bp	[16]
SCC*mec* type IVa	F: GCCTTATTCGAAGAAACCG<br>R: CTACTCTTCTGAAAAGCGTCG	776 bp	[16]
SCC*mec* type IVb	F: TCTGGAATTACTTCAGCTGC<br>R: AAACAATATTGCTCTCCCTC	493 bp	[16]
SCC*mec* type IVc	F: ACAATATTTGTATTATCGGAGAGC<br>R: TTGGTATGAGGTATTGCTGG	200 bp	[16]
SCC*mec* type IVd	F: CTCAAAATACGGACCCCAATACA<br>R: TGCTCCAGTAATTGCTAAAG	881 bp	[16]
SCC*mec* type V	F: GAACATTGTTACTTAAATGAGCG<br>R: TGAAAGTTGTACCCTTGACACC	325 bp	[16]
	**Virulence genes**		
eta	F: ACTGTAGGAGCTAGTGCATTTGT<br>R: TGGATACTTTTGTCTATCTTTTTCATCAAC	190 bp	[58]
etb	F: CAGATAAAGAGCTTTATACACACATTAC<br>R: AGTGAACTTATCTTTCTATTGAAAAACACTC	612 bp	[58]
tst	F: TTCACTATTTGTAAAAGTGTCAGACCCACT<br>R: TACTAATGAATTTTTTTATCGTAAGCCCTT	180 bp	[59]
lukS/F-PV	F: ATCATTAGGTAAAATGTCTGGACATGATCCA<br>R: GCATCAAGTGTATTGGATAGCAAAAGC	443 bp	[60]
